# Impacts of Job Demands on Turnover Intention Among Registered Nurses in Hong Kong Public Hospitals: Exploring the Mediating Role of Burnout and Moderating Effect of Pay Level Satisfaction

**DOI:** 10.1155/2024/3534750

**Published:** 2024-09-16

**Authors:** Ka Po Wong, Bohan Zhang, Yao Jie Xie, Frances Kam Yuet Wong, Claudia Kam Yuk Lai, Shu-Cheng Chen, Jing Qin

**Affiliations:** ^1^ Department of Applied Social Sciences The Hong Kong Polytechnic University, Kowloon, Hong Kong, China; ^2^ School of Nursing The Hong Kong Polytechnic University, Kowloon, Hong Kong, China; ^3^ Faculty of Medicine and Health Sciences Yamaguchi University, Yamaguchi, Japan

**Keywords:** burnout, job demands, job stress, pay level satisfaction, registered nurses, turnover intention

## Abstract

**Background:** High turnover rates and burnout are prevalent issues among registered nurses in public hospitals in Hong Kong. Pay level satisfaction is one of the crucial factors influencing organisational and professional turnover intention. Understanding whether pay level satisfaction can mitigate the negative impact of burnout on turnover intention can provide insights into the role of financial rewards in employee retention.

**Objective:** This study aims to evaluate the relationship between job demands and turnover intention among registered nurses in Hong Kong public hospitals. Additionally, it seeks to examine the mediating role of burnout and explore the potential moderating effect of pay level satisfaction on the relationship between burnout and turnover intention.

**Methods:** The study was a cross-sectional online survey of public hospital staff in Hong Kong. A total of 502 registered nurses who had worked at their employing facility for at least 6 months participated in this cross-sectional survey. Study variables included work overload, job stress, work–family conflict, family–work conflict, conflict with other nurses, burnout, pay level satisfaction and turnover intention. The collected data were analysed using bivariate Pearson correlation analysis and mediated moderation analysis with the PROCESS macro in SPSS 28.0.

**Results:** Burnout mediated the relationship between job demands, including work overload, job stress, work–family conflict, family–work conflict and conflicts with nurses, and organisational and professional turnover intention. Pay level satisfaction did not exert a moderating influence on the relationship between job demands and turnover intention through burnout mediating this relationship.

**Conclusions:** The importance of addressing job stress and burnout to mitigate turnover intention and promote nurse retention is underscored. Contrary to expectations, pay level satisfaction did not buffer the negative impact of job demands on turnover intentions via burnout. This suggests that compensation alone may not be sufficient to offset the detrimental effects of high job demands and burnout on nurses' intention to leave their jobs or the profession. Further research is warranted to explore potential moderators that may influence the relationship between job demands and turnover intention.

## 1. Introduction

Job demands for health have witnessed a significant increase worldwide due to the global phenomenon of rapidly ageing populations and growing healthcare needs [1]. Hong Kong, like many other regions, grapples with the challenges posed by an ageing population, resulting in heightened job demands for nurses [2]. The shortage of nurses is a concern shared by numerous countries, as a growing number of nurses approach retirement age or seek better career opportunities abroad. The aggregate number of registered practicing nurses and fresh nursing graduates grew by over 50%, rising from 43,698 in 2012 to 66,492 in 2022. However, it is projected that there will be shortages of 1383 and 1669 nurses in the years 2025 and 2030, respectively [2].

The healthcare industry is experiencing a shortage of manpower, coupled with escalating work demands, which has resulted in healthcare professionals working long hours and facing overwhelming workloads. Consequently, this has led to burnout and physical and mental exhaustion among healthcare professionals, ultimately increasing their intention to leave their positions [3]. The high turnover intention rate of registered nurses can have detrimental effects on the quality of patient care and the overall functioning of the healthcare system [1]. These repercussions include a lack of continuity of care for patients, increased workload in nurses who stayed, limitation of time nurses spending on each patient, increased healthcare cost, negative impact on employee morale and challenges in recruitment and staff orientation and training.

While there is a substantial body of literature on job demands, burnout and turnover intervention among healthcare practitioners, there is a notable gap in research focussing on the context of registered nurses within the Hong Kong public hospital setting. Previous studies, which are not limited to a specific geographical context, mainly focused on the intention to leave the profession. Little is known about the impact of job demands on the intention to leave the hospital. Existing research underexplored the moderating role of pay level satisfaction in the relationship between job demands, burnout and turnover intention. No cross-sectional study has been conducted to examine the direct and indirect effects of job demands on intention to leave hospitals and professions in registered nurses working in Hong Kong public hospitals. Hence, an empirical study is needed to explore the moderating effect of pay level satisfaction amongst registered nurses and provide valuable insights and potential solutions that can be adapted and implemented in healthcare systems facing similar workforce challenges. The findings of this study have the potential to inform policies and interventions aimed at improving nurse retention and enhancing the quality of patient care, not only in Hong Kong but also in other healthcare settings worldwide.

## 2. Background

### 2.1. Nexus of Job Demands, Burnout and Turnover Intention in Nursing

The nursing profession is grappling with a global challenge of elevated job demands, which have been identified as a significant factor contributing to burnout and turnover intention among employees. Job demands encompass the physical, mental and emotional efforts required to perform a job [4]. In the context of Hong Kong, registered nurses in public hospitals are particularly affected by high-demand workloads, as they are tasked with providing care for a large volume of patients [5]. Despite the international recommendation of 9 nurses per 1000 people, Hong Kong falls slightly short with a ratio of 8.2 nurses per 1000 individuals [2, 6]. This shortfall contributes to excessive workloads, adherence to strict shift schedules, and the need for nurses to engage in training programs outside regular working hours [7, 8]. Factors such as long working hours, heavy workloads, inadequate staffing, and a lack of support for professional development have been cited as reasons for occupational turnover among nurses in Hong Kong [9].

The phenomenon of burnout and turnover intention is not unique to Hong Kong but is also observed in other regions. For instance, studies in China have shown a positive relationship between role demands and the intention to leave the hospital among frontline nurses [10], while South Korean nurses have demonstrated a positive association between burnout, emotional exhaustion and turnover intention [11]. Work–family conflict, where work responsibilities hinder the ability to meet family obligations, and family–work conflict, where family obligations impede work responsibilities, have both been positively associated with turnover intention [12–14]. Other studies have identified factors such as heavy workloads, relationships with physicians, full-time work schedules, burnout, and participation in hospital affairs as contributors to nurses' intention to leave the profession [15–17].

Burnout, a psychological response to chronic work stress, can mediate the relationship between job stress and turnover intention [18, 19]. It arises from continuous exposure to high patient volumes, complex medical cases, and emotionally demanding situations, leading to emotional exhaustion and detachment [20]. The mediating role of burnout on the impacts of job demands and family–work conflict on turnover intention has been demonstrated in various studies [21–24]. In addition to the direct impact of workload, nurses also face administrative tasks and interpersonal conflicts that can exacerbate job stress and reduce job satisfaction, potentially leading to turnover intention [25–27]. The implications of turnover intention are profound, affecting not only the healthcare system and continuity of patient care but also the well-being of nurses themselves [28]. Therefore, understanding and addressing the multifaceted nature of job demands is crucial for enhancing retention in the nursing profession, particularly in the high-pressure environment of Hong Kong's public hospitals.

### 2.2. Effect of Pay Levels Satisfaction on Nurses

Pay level satisfaction, which reflects an individual's subjective evaluation of their job compensation, has been recognised as a potential buffer against the negative effects of high job demands and burnout on nurses' turnover intentions [29, 30]. Studies across various regions have demonstrated the importance of pay satisfaction in the nursing profession. Wang et al. [31] found that pay satisfaction influenced geriatric nurses' turnover intentions both directly and indirectly. In China, Wang et al. [32] reported that wage satisfaction was a critical component of job satisfaction that negatively correlated with turnover intentions among primary care providers. The finding of a negative relationship between pay satisfaction and turnover intention echoes the results reported by Wang et al. [31] in their investigation of geriatric nursing personnel in China. Internationally, Wardhani and Hariyati's [33] qualitative research in Indonesia revealed that pay growth played a significant role in nurse retention. High pay satisfaction may alleviate emotional exhaustion, a key aspect of burnout, thereby enhancing the ability to manage job demands and reducing the likelihood of turnover [34, 35]. Despite these insights, the moderating role of pay level satisfaction in the relationship between burnout and turnover intention remains underexplored. This study aims to fill this gap by examining whether pay level satisfaction can act as a counterbalance to the adverse effects of job demands and burnout on turnover intention, with a particular focus on the conditions faced by registered nurses in Hong Kong's public hospitals.

### 2.3. Theoretical Framework

**Figure 1:**
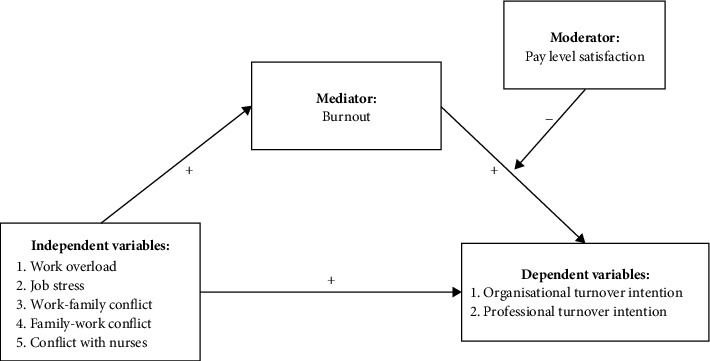
Study conceptual model.

The conceptual framework of this study (see Figure 1) was built upon the job demands-resources (JD-R) model proposed by Bakker and Demerouti [34]. This model suggests the interaction between job demands and job resources, where job demands are associated with negative effects on employee well-being, while job resources are associated with positive effects on employees [36]. Based on this model to explain the conceptual framework of this study, job demands refer to the physical and mental effort involved in work-related aspects. Specifically, job demands in our study include workload, job stress, work–family conflict, family–work conflict and conflict with nurses. These factors have been identified as significant dimensions within our research context and are crucial for understanding their impact on employee well-being and other outcome variables [4]. Burnout is the consequence of prolonged exposure to job demands and is the result of the imbalance between job demands and available resources [20]. The conceptual framework of this study proposes burnout as a mediator between job demands-related factors and turnover intention, while pay level satisfaction acts as a moderating factor. The study hypothesises that work overload, job stress, work–family conflict, family–work conflict and conflict with nurses will have direct positive effects on turnover intention and an indirect positive effect through burnout. Additionally, we postulated that pay level satisfaction will moderate the relationship between job demands-related factors, burnout, and organisational and professional turnover intention.

This study aimed to examine the conditional indirect effects of workload, job stress, work–family conflict, family–work conflict and conflict with nurses on organisational and professional turnover intention, with burnout serving as the mediator and to explore the moderating influence of pay level satisfaction in this relationship. This investigation was conducted using a two-step approach: (1) assessing whether the relationship between five job demands-related factors and organisational and professional turnover intention is mediated by burnout (i.e., mediation model) and (2) examining whether the strength of the mediated relationship is moderated by pay level satisfaction (i.e., mediated moderation analysis). More specifically, it was hypothesised that the five components of job demands, namely, work overload, job stress, work–family conflict, family–work conflict and conflict with nurses, would have a positive direct effect on organisational and professional turnover intention. Additionally, it was hypothesised that burnout would mediate the relationship between the five job demands-related factors and turnover intention, exerting an indirect positive effect. It was asserted that pay level satisfaction would moderate the mediated relationship. Particularly, it was hypothesised that the direct and indirect relationship between the five job demands-related factors and organisational and professional turnover intention would be weaker when there was a high level of pay level satisfaction.

## 3. Methods

### 3.1. Design and Participants

The present study adopted a cross-sectional approach. Data were collected from registered nurses working at public hospitals in Hong Kong between October and December 2022. An online survey was randomly sent to the hospital executives or managers of the 43 public hospitals in Hong Kong. Ultimately, 13 public hospitals accepted the invitation. The purposes and the contents of the survey were demonstrated in the email and the first page of the online survey. The managers were asked to forward the online survey link to the nurses if they consented to be involved after reading the materials we sent. Participants' consent was confirmed by agreeing, completing and submitting the online survey. The online survey was made anonymous to preserve the confidentiality of the subjects' identities. Approval for the study was obtained from the Institutional Review Board of the authors' university (application number: HSEARS20220602001).

To be eligible for this study, participants should be (i) a registered nurse; (ii) currently working in a public hospital for at least 6 months; (iii) between the ages of 18 and 65; and (iv) able to read and write Chinese. Registered nurses who have worked in public hospitals for 6 months or more seem to be more familiar with the workings of the public healthcare system.

### 3.2. Measures

Job demands comprised five elements, including work overload, job stress, work–family conflict, family–work conflict and conflict with nurses. All items in the questionnaire were assessed by a seven-point response scale ranging from 1 (strongly disagree) to 7 (strongly agree). Higher scores indicated a higher level of job stress. Work overload was assessed by a five-item scale derived from Reilly's Role Overload Scale (*α* = 0.66–0.87). The scale has been validated and widely applied [37]. Job stress is measured by four items adapted from the study of Graham et al. [38]. These items have been widely applied in assessing job stress amongst healthcare professionals [39, 40]. The Work–Family Conflict Scale (WAFCS), created by Haslam et al. [41], was the instrument used to gauge five work-to-family conflict items and five family-to-work conflict items. The measure had a high internal consistency (work–family conflict: *α* = 0.84) and (family–work conflict: *α* = 0.80). Conflict with nurses was assessed by five items adapted from the Nursing Stress Scale developed by Gray-Toft and Anderson [42] (*α* = 0.7).

Burnout was measured using six items adapted from the Maslach Burnout Inventory [43]. These six items were measured with a seven-point Likert scale which ranged from 1 (completely disagree) to 7 (completely agree). A higher rating indicated a higher level of burnout. This scale has been validated by Demerouti, Mostert and Bakker [44] and had a good internal consistency (*α* = 0.82).

Pay level satisfaction was measured using five items adapted from Smith, Kendall, and Hulin [45] and Hackman and Oldham [46]. These five items were measured with a seven-point Likert scale which ranged from 1 (completely disagree) to 7 (completely agree). High scores indicated a high level of pay level satisfaction. The scale has a good internal consistency (*α* = 0.83).

Turnover intention was assessed using two items, including “I do not want to continue working in this organisation” and another one was “I do not want to do this career.” These two items were adapted from the study of Yamaguchi et al. [47]. These two items were measured with a seven-point Likert scale which ranged from 1 (completely disagree) to 7 (completely agree). High scores indicated higher turnover intention.

A series of questions about demographic information such as age, gender, educational level, position and number of years since registration were included.

### 3.3. Data Analysis

The measurement model was verified through confirmatory factor analysis (CFA) using SPSS 28.0 and AMOS 28.0. CFA was conducted on the measurement model using SPSS 28.0 and AMOS 28.0 to assess its validity. Six goodness-of-fit indices were employed, namely, chi square to degree of freedom (*χ*^2^/ d*f*), goodness-of-fit index (GFI), incremental fit index (IFI), comparative fit index (CFI), Tucker–Lewis Index (TLI), root mean square error of approximation (RMSEA), Parsimony goodness-of-fit index (PGFI) and average variance extracted (AVE) [48]. The statistics analysis was performed using SPSS 28.0. The demographics of the participants were presented in descriptive analysis. The correlations between the participants' demographics and study variables (job demands, burnout, pay level satisfaction and turnover intention) were examined by the bivariate Pearson correlation coefficient. The mediated moderation analyses were examined by PROCESS macro by Hayes [49]. The unstandardised coefficient beta and standard error for each path (between the study variables) were presented. This PROCESS macro tests the direct and indirect relationship between job demands and turnover intention and examines the mediator of burnout and the moderator of pay level satisfaction in this relationship. If significant moderating effects of pay level satisfaction were found, further moderating effects on different levels of pay satisfaction on turnover intention would be tested.

## 4. Results

### 4.1. Participant Characteristics

Table 1 demonstrates the description of participant characteristics. A total of 502 completed questionnaires were received from 13 hospitals. The study sample was comprised entirely of full-time registered nurses. Most of the participants were female (71.3%). The largest age group was those aged 30 to 40 (39.6%). 52.0% of the participants were single. 97.6% of the participants had a bachelor's degree or above. The average year registered as a registered nurse was 13.1 (SD: 10.1) years. The average working hours per week was 46.5 (SD: 7.0) hours.

### 4.2. Results of Validity and Reliability of the Measurement Model

Table 2 shows the results of the validity and CFA of the measurement model. The values of AVE of all constructs ranged from 0.50 to 0.70 suggesting a good level of convergent validity. The values of *χ*^2^/d*f*, GFI, IFI, CFI, TLI, RMSEA and PGFI were 2.88 (*p* < 0.001), 0.92, 0.95, 0.95, 0.92, 0.06 and 0.70, respectively, satisfied with statistical standards [48]. Therefore, the measurement model presented satisfactory fit indices.

### 4.3. Correlations of Variables

The Spearman correlation coefficient was used since the data set did not exhibit a normal distribution according to the Shapiro–Wilk test.  Table 3 presents the Spearman correlation between the variances. Work overload, job stress, work–family conflict, conflict with nurses and burnout were positively correlated with organisational and professional turnover intention. Pay level satisfaction was negatively correlated with organisational and professional turnover intention. There was no significant association found between nurses' experiences of family-interfering-with-work conflict and their reported plans to leave their current job or the nursing profession. Work overload, job stress, work–family conflict, family–work conflict and conflict with nurses were positively correlated with burnout. Work overload, job stress, work–family conflict, conflict with nurses and burnout were negatively correlated with pay level satisfaction and family–work conflict had no significant correlation with pay level satisfaction.

### 4.4. Mediated Moderation Analysis

Work overload, job stress, work–family conflict, family–work conflict and conflict with other nurses has a positive statistically significant impact on burnout (work overload: *B* = 0.590, *p* < 0.001; job stress: *B* = 0.817, *p* < 0.001; work–family conflict: *B* = 0.581, *p* < 0.001, family–work conflict: *B* = 0.256, *p* < 0.001; conflict with nurses: *B* = 0.480, *p* < 0.001) (Table 4).

Table 5 demonstrates the results of mediated moderation analysis of burnout and pay level satisfaction on the relationship between job demands-related factors and organisational turnover intention. Statistically significant associations were found between work overload (*B* = 0.153, *p* < 0.028), job stress (*B* = 0.287, *p* < 0.014) and conflict with other nurses (*B* = 0.280, *p* < 0.001) with organisational turnover intention. Additionally, family–work conflict (*B* = 0.574, *p* < 0.001) was positively associated with organisational turnover intention, while work–family conflict did not demonstrate a significant effect. Burnout was found to have statistically significant mediating effects in all five relationships (*p* < 0.001). Pay level satisfaction had a statistically significant impact on organisational turnover intention (*p* < 0.001). However, pay level satisfaction did not demonstrate a significant moderating effect on the mediated relationship.

The results of the mediated moderation analysis, examining the influence of pay level satisfaction as a moderator on the relationship between job demands-related factors, burnout and professional turnover intention are demonstrated in Table 5. Work overload (*B* = 0.253, *p* < 0.002) and conflict with other nurses (*B* = 0.351, *p* < 0.001) showed statistically significant associations with professional turnover intention, while job stress, work–family conflict and family–work conflict did not exhibit a significant effect. Burnout was found to have statistically significant mediating effects in all five relationships (*p* < 0.001). Furthermore, pay level satisfaction showed a statistically significant impact on organisational turnover intention (*p* < 0.05). However, pay level satisfaction did not demonstrate a significant moderating effect on the mediated relationship.

Table 6 shows the results of the direct and conditional indirect effects of job demands-related factors on organisational turnover intention with the mediator of burnout and the moderator of pay level satisfaction. For the indirect effects of work overload, the indirect effect in the presence of the moderator, pay level satisfaction, at mean level, one standard deviation below mean and above mean were 0.333, 0.304 and 0.362, respectively, and per the bootstrap, that is within the confidence interval at a *p* < 0.05. Similarly, for job stress, the bootstrap analysis revealed significant effects at mean level, one standard deviation below mean, and one standard deviation above mean of pay level satisfaction (0.419, 0.395 and 0.442, respectively, *p* < 0.05). Work–family conflict also showed significant indirect effects on organisational turnover intention at the different levels of pay level satisfaction (0.333, 0.311 and 0.356, respectively, *p* < 0.05). The indirect effects of family–work conflict on organisational turnover intention were significant at mean level, one standard deviation below mean and one standard deviation above mean of pay level satisfaction (0.172, 0.158 and 0.187, respectively, *p* < 0.05). Conflict with other nurses had significant indirect effects on organisational turnover intention across the different levels of pay level satisfaction (0.228, 0.208 and 0.248, respectively, *p* < 0.05). The findings suggest indirect effect of work overload, job stress, work–family conflict, family–work conflict and conflict with other nurses on organisational turnover intention through burnout is stronger or more pronounced when registered nurses have a higher level of satisfaction with their pay levels. The index of mediated moderation is not significant, indicating that the indirect effect is not moderated by pay level satisfaction.

Table 6 presents the results of direct and conditional indirect effects of job demands-related factors on professional turnover intention. Burnout serves as the mediator, and pay level satisfaction acts as the moderator. Considering the indirect effects of different factors on professional turnover intention, the bootstrap analysis shows that work overload has a significant indirect effect in the presence of pay level satisfaction at mean level, one standard deviation below mean and one standard deviation above mean (0.293, 0.277 and 0.309, respectively, *p* < 0.05). Similarly, job stress has a significant indirect effect on professional turnover intention across the different levels of pay level satisfaction (0.434, 0.429 and 0.439, respectively, *p* < 0.05). Work–family conflict demonstrates a significant indirect effect on professional turnover intention at mean level, one standard deviation below mean and one standard deviation above mean of pay level satisfaction (0.308, 0.302 and 0.315, respectively, *p* < 0.05). Likewise, family–work conflict shows a significant indirect effect on professional turnover intention across the different levels of pay level satisfaction (0.165, 0.159 and 0.172, respectively, *p* < 0.05). Moreover, conflict with other nurses has a significant indirect effect on professional turnover intention in the presence of different levels of pay level satisfaction (0.199, 0.192 and 0.206, respectively, *p* < 0.05). The findings suggest that indirect effect of work overload, job stress, work–family conflict, family–work conflict and conflict with other nurses on professional turnover intention through burnout is stronger or more pronounced when registered nurses have a higher level of satisfaction with their pay levels. The index of mediated moderation is not significant, indicating that the indirect effect is not moderated by pay level satisfaction.

## 5. Discussion

The present study's exploration of the mediating role of burnout in the relationship between work overload, job stress, work–family conflict, family–work conflict, conflict with nurses, and organisational and professional turnover intentions among registered nurses in Hong Kong's public hospitals has yielded significant insights. We can gain a better understanding of the complex interplay between job demands-related factors and burnout and turnover intentions, thereby enhancing our knowledge of the challenges faced by nurses and the potential strategies for improving retention in the nursing profession by delving deeper into the results.

Our findings highlight the mediating role of burnout in the relationship between the five factors of job demands and organisational and professional turnover intentions. These are consistent with previous studies that determined burnout as the response to job demand, job stress, work overload, work–family conflict, family–work conflict and poor relationship with colleagues [18, 50–52]. Moreover, our findings confirm that the intention to leave both the current job and the nursing profession can be attributed to burnout among nurses [15, 22].

The study revealed significant effects of work overload, job stress and conflicts with nurses on organisational turnover intention. Work overload and conflicts with nurses were found to have significant effects on professional turnover intention. Remarkably, conflict with other nurses emerged as the primary and most prominent correlate, influencing both organisational and professional turnover intentions. These findings match the earlier studies that workloads are the driving factors of nurses' organisational and professional turnover intentions [12, 53]; nurses' job stress leads to the intention to leave the hospitals [54, 55]; conflict with colleagues increases the intention to leave the hospitals and occupation [54, 56]. Reinhardt et al. [57] found that better relationships with colleagues improved retention amongst registered nurses in the United States. These results corroborate the established understanding that burnout acts as a critical mediator between job demands-related factors and organisational as well as professional turnover intentions, aligning with the theoretical framework posited by the JD-R model [34].

Contrary to previous studies [29, 58], pay level satisfaction did not moderate the relationships between job demands-related factors, burnout, and organisational and professional turnover intentions. These unexpected results may be due to uniform pay structure, perceived fairness of pay systems and policies, nonfinancial factors and cultural norms and values. A standardised salary scale for registered nurses may limit variability in pay levels across the labour [59]. There may not be enough variation in pay level satisfaction to detect a significant moderating effect when most registered nurses receive similar pay under this pay system. A transparent pay system may lead to perceptions of fairness and pay equity [59–61]. This may diminish the moderating effect of pay level satisfaction on the job demands-turnover intention relationship. Further, nonfinancial factors, such as job autonomy, supportive work environments or professional development opportunities, which may be more influential in determining nurses' turnover intention among nurses, might overshadow the impact of pay level satisfaction as a moderator [62]. Cultural norms and values, such as a strong work ethic, could be a prioritising factor in determining the intention to leave [63, 64].

According to the JD-R model, pay level satisfaction is expected to buffer the negative effects of job demands on turnover intention [65]. However, if the employee perceived their job demands as compensated by adequate pay, the moderating effect of pay level satisfaction might be diminished. In this case, the nurses may perceive other job resources, such as social support and career development opportunities, as compensation for high job demands [66]. For instance, the meta-analysis of Kim and Kim [11] showed that factors other than salary, including interpersonal relationships, social support, organisational justice and person–organisational fit, were significantly and negatively associated with turnover intention amongst nurses in South Korea. As a result, the moderating effect of pay level satisfaction may be attenuated. The specific reasons for the lack of moderation effect of pay level satisfaction in the given context would require empirical investigation and further research to provide more concrete explanations.

It is somewhat surprising that family–work conflict had negative impacts on organisational and professional turnover intention, while work–family conflict had no significant association with organisational and professional turnover intention. Extensive research has shown that work–family conflict and family–work conflict predict the nurses' intention to leave the hospitals and profession [47, 67, 68]. This finding may reflect a complex interplay of personal and professional values, cultural expectations and life-stage considerations that influence turnover decisions. It is possible that for the younger, predominantly single nurses in this study, professional aspirations and career progression may take precedence over family obligations, thus reducing the impact of family–work conflict on their turnover intentions. Our demographic analysis revealed that younger caregivers in our study (under 30 years old) were more likely to be single and without dependent children. This suggests that family obligations may be less important for this group than for older, married/partnered individuals. Further qualitative research could provide richer insights into these personal and cultural factors that shape nurses' work-life integration and turnover behaviour.

Regarding practical implications, this study highlights the significance of addressing conflict with other nurses. Conflict with colleagues emerged as the primary and most prominent factor in organisational and professional turnover intention. Healthcare organisations should prioritise interventions aimed at promoting effective communication, collaboration and conflict resolution among nursing staff. Creating a positive work environment that fosters teamwork and mutual respect can help mitigate conflicts and reduce turnover intentions. Furthermore, the study underscores the importance of managing work overload. Healthcare organisations should explore strategies to effectively manage workloads, such as workload redistribution, task prioritisation and resource allocation. By addressing work overload, healthcare organisations can alleviate the burden on nurses and enhance job satisfaction, ultimately reducing turnover intentions. Additionally, the findings emphasise the role of burnout as a mediating factor. The study identified statistically significant mediating effects of burnout in all five of the relationship dynamics that were explored. To mitigate burnout among nursing staff, healthcare organisations should prioritise interventions aimed at preventing and managing burnout. This may include providing resources for stress reduction, promoting self-care practices and offering emotional support to nurses. By addressing burnout, healthcare organisations can improve job satisfaction and reduce turnover intentions.

This research further accentuates how family-interfering-with-work conflict can shape nurses' considerations of quitting their organisation. Healthcare organisations should recognise and address the challenges faced by nurses in balancing their work and family responsibilities. Implementing family–friendly policies, flexible work arrangements and support systems for childcare or eldercare can help reduce family–work conflict and mitigate turnover intentions among nursing staff. However, it is important to note that pay level satisfaction did not demonstrate a significant moderating effect on the relationship between job demands-related factors and burnout and turnover intentions. Therefore, healthcare organisations should adopt a comprehensive approach that considers various factors, including work environment, workload management, interpersonal relationships and support systems, in addition to fair compensation. This multifaceted approach should go beyond salary adjustments and encompass strategies such as mentorship programs, career development pathways and wellness initiatives that target burnout prevention. By considering these multiple factors, organisations can create an environment that supports nurses' well-being and reduces the likelihood of turnover. Taking a holistic approach to nurse retention will foster a positive work environment and promote the long-term satisfaction and commitment of registered nurses in the healthcare sector.

### 5.1. Limitations and Future Research Directions

This study contains several limitations. First, the cross-sectional design limited the ability to establish causal relationships between study variables. To provide more robust evidence of causality by capturing changes in variables over time, future research is encouraged to employ longitudinal designs. This approach would enable a deeper understanding of the temporal dynamics and causal pathways involved. Second, it is important to note that the participants in this study consisted of registered nurses from public hospitals in Hong Kong. Thus, caution should be exercised when generalising the findings to other contexts, such as private hospitals or healthcare systems in different countries. Further investigations should encompass diverse healthcare settings and populations to enhance the generalisability and external validity of the results. Third, the present study did not include certain variables that may exert influence on the examined relationships. Variables such as organisational culture, social support, and job autonomy are recognised as potential factors in the complex interplay between work-related elements and burnout and turnover intentions. Incorporating these additional variables in future research endeavours would yield a more comprehensive understanding of the phenomenon and enable the identification of additional factors that contribute to the outcomes of interest.

To advance the current knowledge in this field, future research should explore multiple avenues. First, employing longitudinal designs would aid in understanding the long-term effects of work-related factors on burnout and turnover intentions, as well as uncovering causal mechanisms. Second, including a wider range of variables such as organisational culture, social support, job autonomy, and other relevant psychosocial factors would provide a more comprehensive understanding of the complexities involved. Additionally, expanding the investigation to encompass different healthcare contexts, both nationally and internationally, would enhance the applicability and external validity of the findings. Moreover, qualitative research methods, such as interviews or focus groups, could be used to gain in-depth insights into nurses' experiences and perspectives concerning work-related factors and burnout and turnover intentions. These qualitative approaches would complement the quantitative findings and offer a deeper understanding of the underlying mechanisms.

## 6. Conclusions

The study was to determine the mediating effect of burnout and the moderating effect of pay level satisfaction in the indirect effect of job demands on turnover intention. The findings highlight the detrimental impact of work-related factors, such as work overload, job stress and conflict with other nurses, on nurse burnout and turnover intentions. While pay level satisfaction was negatively correlated to turnover intention, the lack of a significant moderating effect suggests that addressing burnout and turnover intentions requires a comprehensive approach that goes beyond focussing solely on pay satisfaction. Public hospitals should consider implementing strategies that tackle multiple factors contributing to burnout. The findings emphasise the need for healthcare organisations and policymakers to prioritise interventions aimed at reducing work-related stress and burnout among registered nurses in public hospitals in Hong Kong. By addressing these factors and fostering a supportive work environment, healthcare organisations can promote nurse well-being, mitigate turnover intentions and contribute to the overall quality of patient care.

## Figures and Tables

**Table 1:** Descriptive statistics of sample demographics and key variables (*N* = 502).

**Nurse demographics**		**Frequency (%)**	**Mean (SD)**	**Range**
Age	≤30	133 (26.5)	—	—
	>30–40	199 (39.6)	—	—
	>40–50	92 (18.3)	—	—
	>50	78 (15.5)	—	—

Gender	Male	144 (28.7)	—	—
	Female	358 (71.3)	—	—

Marital status	Single	261 (52.0)	—	—
	Married	234 (46.6)	—	—
	Divorced	7 (1.4)	—	—

Education	Diploma/higher diploma	12 (2.4)	—	—
	Bachelor's degree	201 (40.0)	—	—
	Master's degree	289 (57.6)	—	—

Years since registration		—	13.1 (10.1)	1.0–40.0

Years of working in this unit		—	7.8 (7.4)	0.4–35.0

Work departments	Cardiology	28 (5.6)	—	—
	Infectious disease department	11 (2.2)	—	—
	Intensive care unit	43 (8.6)	—	—
	Medical department	161 (32.1)	—	—
	Neurosurgery	2 (0.4)	—	—
	Obstetrics and gynaecology	53 (10.6)	—	—
	Oncology department	19 (3.8)	—	—
	Orthopaedics and traumatology	39 (7.8)	—	—
	Otolaryngology	11 (2.2)	—	—
	Paediatrics	54 (10.8)	—	—
	Surgery	38 (7.65)		
	Others	43 (8.6)	—	—

Position	Registered nurse	335 (66.7)	—	—
	Advanced practice nurse/nursing officer	167 (33.3)	—	—

Average working hours per weeks (hours)		—	46.5 (7.0)	35–78

Need to raise children		174 (34.7)	—	—

No need to raise children		328 (65.3)	—	—

Need to take care of family members (in addition to children)		237 (47.2)	—	—

No need to take care of family members (in addition to children)		265 (52.8)	—	—

Living with family		453 (90.2)	—	—

Not living with family		49 (9.8)	—	—

Abbreviation: SD, standard deviation.

**Table 2:** Validity of the measurement model.

**Construct**	**Item**	**Factor loading**	**Average variance extracted**	**Composite reliability**
Job demands	Work overload	0.69	0.50	0.83
	Job stress	0.70		
	Work–family conflict	0.81		
	Family–work conflict	0.78		
	Conflict with other nurses	0.51		

Burnout	Burnout 1	0.78	0.64	0.91
	Burnout 2	0.82		
	Burnout 3	0.77		
	Burnout 4	0.88		
	Burnout 5	0.83		
	Burnout 6	0.70		

Pay level satisfaction	Pay level satisfaction 1	0.85	0.70	0.92
	Pay level satisfaction 2	0.77		
	Pay level satisfaction 3	0.87		
	Pay level satisfaction 4	0.86		
	Pay level satisfaction 5	0.83		

Turnover intention	Organisational turnover intention	0.83	0.69	0.81
	Professional turnover	0.82		

**Table 3:** Spearman correlation of study variables.

	**1**	**2**	**3**	**4**	**5**	**6**	**7**	**8**	**9**	**10**
1. Average working hours per week	1.000									
2. Work overload	0.302^∗∗^	1.000								
3. Job stress	0.311^∗∗^	0.586^∗∗^	1.000							
4. Work–family conflict	0.352^∗∗^	0.531^∗∗^	0.555^∗∗^	1.000						
5. Family–work conflict	0.183^∗∗^	0.202^∗∗^	0.214^∗∗^	0.413^∗∗^	1.000					
6. Conflict with other nurses	0.111^∗^	0.386^∗∗^	0.322^∗∗^	0.438^∗∗^	0.290^∗∗^	1.000				
7. Burnout	0.201^∗∗^	0.495^∗∗^	0.621^∗∗^	0.639^∗∗^	0.227^∗∗^	0.504^∗∗^	1.000			
8. Pay level satisfaction	−0.123^∗∗^	−0.133^∗∗^	−0.401^∗∗^	−0.286^∗∗^	−0.045	−0.171^∗∗^	−0.354^∗∗^	1.000		
9. Organisational turnover intention	0.162^∗∗^	0.327^∗∗^	0.481^∗∗^	0.411^∗∗^	0.061	0.409^∗∗^	0.557^∗∗^	−0.357^∗∗^	1.000	
10. Professional turnover intention	0.028	0.339^∗∗^	0.376^∗∗^	0.363^∗∗^	0.077	0.394^∗∗^	0.454^∗∗^	−0.245^∗∗^	0.567^∗∗^	1.000

^∗∗^ *p* < 0.01, ^∗^*p* < 0.05.

**Table 4:** Mediation analysis of burnout with work overload, job stress, work–family conflict, family–work conflict and conflict with other nurses.

**Predictors**	**Burnout (M)**	**95% CI**
	**B (SE) (95% CI)**	
Intercept	−3.219^∗∗^ (0.236)	−3.682, −2.755
Work overload	0.590 ^∗∗^ (0.425)	0.507, 0.674
	*R* ^2^ = 0.279 *F* = 193.248, *p* < 0.001	
Intercept	−4.871^∗∗^ (0.259)	−5.379, −4.363
Job stress	0.817^∗∗^ (0.043)	0.733, 0.902
	*R* ^2^ = 0.421 *F* = 363.731, *p* < 0.001	
Intercept	−3.100^∗∗^ (0.163)	−3.419, −2.780
Work–family conflict	0.581^∗∗^ (0.030)	0.523, 0.639
	*R* ^2^ = 0.436 *F* = 385.686, *p* < 0.001	
Intercept	−0.933^∗∗^ (0.143)	−1.213, −0.652
Family–work conflict	0.256^∗∗^ (0.036)	0.184, 0.328
	*R* ^2^ = 0.089 *F* = 48.833, *p* < 0.001	
Intercept	−1.672^∗∗^ (0.128)	−1.923, −1.421
Conflict with other nurses	0.480^∗∗^ (0.034)	0.413, 0.548
	*R* ^2^ = 0.282 *F* = 196.168, *p* < 0.001	

Abbreviations: CI, confidence interval; M, mediator; SE, standard error.

^∗∗^ *p* < 0.001.

**Table 5:** Mediated moderation analysis of burnout and pay level satisfaction on the relationship between work overload, job stress, work–family conflict, family–work conflict and conflict with other nurses, and organisational and professional turnover intentions.

**Predictors**	**B (SE)**	**95% CI**
*Organisational turnover intention (Y)*		
Intercept	3.810^∗∗^ (0.383)	3.057, 4.562
Work overload	0.153^∗^ (0.069)	0.017, 0.289
Burnout	0.565^∗∗^ (0.068)	0.431, 0.699
Pay level satisfaction	−0.282^∗∗^ (0.048)	−0.377, −0.187
Burnout ^*∗*^ pay level satisfaction	0.035 (0.039)	−0.041, 0.112
	*R* ^2^ = 0.336 *F* = 62.727, *p* < 0.001	
Intercept	2.925^∗∗^ (0.536)	1.872, 3.979
Job stress	0.287^∗^ (0.089)	0.112, 0.462
Burnout	0.512^∗∗^ (0.071)	0.373, 0.651
Pay level satisfaction	−0.240^∗∗^ (0.049)	−0.337, −0.143
Burnout ^*∗*^ pay level satisfaction	0.021 (0.039)	−0.056, 0.097
	*R* ^2^ = 0.343 *F* = 64.788, *p* < 0.001	
Intercept	4.134^∗∗^ (0.339)	3.468, 4.801
Work–family conflict	0.094 (0.062)	−0.028, 0.216
Burnout	0.574^∗∗^ (0.073)	0.430, 0.718
Pay level satisfaction	−0.268^∗∗^ (0.049)	−0.364, −0.173
Burnout ^*∗*^ pay level satisfaction	0.028 (0.039)	−0.049, 0.104
	*R* ^2^ = 0.332 *F* = 61.781, *p* < 0.001	
Intercept	4.995^∗∗^ (0.189)	4.623, 5.367
Family–work conflict	−0.096^∗^ (0.048)	−0.190, 0.018
Burnout	0.673^∗∗^ (0.061)	0.553, 0.792
Pay level satisfaction	−0.270^∗∗^ (0.048)	−0.365, −0.175
Burnout ^*∗*^ pay level satisfaction	0.041 (0.039)	−0.036, 0.118
	*R* ^2^ = 0.334 *F* = 62.415, *p* < 0.001	
Intercept	3.665^∗∗^ (0.203)	3.267, 4.063
Conflict with other nurses	0.280^∗∗^ (0.055)	0.172, 0.388
Burnout	0.475^∗∗^ (0.066)	0.345, 0.605
Pay level satisfaction	−0.282^∗∗^ (0.047)	−0.375, −0.189
Burnout ^*∗*^ pay level satisfaction	0.030 (0.038)	−0.045, 0.104
	*R* ^2^ = 0.362 *F* = 70.562, *p* < 0.001	

*Professional turnover intention (Y)*		
Intercept	2.501^∗∗^ (0.453)	1.611, 3.392
Work overload	0.253^∗^ (0.082)	0.092, 0.415
Burnout	0.497^∗∗^ (0.081)	0.339, 0.655
Pay level satisfaction	−0.160^∗^ (0.057)	−0.269, −0.044
Burnout ^*∗*^ pay level satisfaction	0.019 (0.046)	−0.071, 0.110
	*R* ^2^ = 0.218 *F* = 34.708, *p* < 0.001	
Intercept	2.651^∗∗^ (0.642)	1.404, 3.858
Job stress	0.205 (0.107)	0.004, 0.414
Burnout	0.531^∗∗^ (0.085)	0.365, 0.698
Pay level satisfaction	−0.121^∗^ (0.059)	−0.237, −0.052
Burnout ^*∗*^ pay level satisfaction	0.046 (0.046)	−0.087, 0.957
	*R* ^2^ = 0.209 *F* = 32.888, *p* < 0.001	
Intercept	3.177^∗∗^ (0.403)	2.384, 3.969
Work–family conflict	0.131 (0.074)	−0.014, 0.276
Burnout	0.531^∗∗^ (0.087)	0.360, 0.702
Pay level satisfaction	−0.137^∗^ (0.058)	−0.250, −0.023
Burnout ^*∗*^ pay level satisfaction	0.008 (0.046)	−0.083, 0.099
	*R* ^2^ = 0.208 *F* = 32.713, *p* < 0.001	
Intercept	4.129^∗∗^ (0.226)	3.685, 4.572
Family–work conflict	−0.068 (0.057)	−0.180, 0.044
Burnout	0.646^∗∗^ (0.073)	0.503, 0.789
Pay level satisfaction	−0.143^∗^ (0.058)	−0.257, −0.030
Burnout ^*∗*^ pay level satisfaction	0.019 (0.047)	−0.073, 0.111
	*R* ^2^ = 0.206 *F* = 32.169, *p* < 0.001	
Intercept	2.654^∗∗^ (0.240)	2.182, 3.126
Conflict with other nurses	0.351^∗∗^ (0.065)	0.223, 0.479
Burnout	0.415^∗∗^ (0.078)	0.261, 0.569
Pay level satisfaction	−0.154^∗∗^ (0.056)	−0.265, −0.044
Burnout ∗ pay level satisfaction	0.010 (0.045)	−0.078, 0.099
	*R* ^2^ = 0.247 *F* = 40.820, *p* < 0.001	

Abbreviations: CI, confidence interval; M, mediator; SE, standard error; Y, outcome variable.

^∗∗^ *p* < 0.001, ^∗^*p* < 0.05.

**Table 6:** Direct and conditional indirect effects of work overload, job stress, work–family conflict, family–work conflict and conflict with other nurses on the organisational and professional turnover intention with the mediator of burnout and moderator of pay level satisfaction.

**Relationship**	**Direct effect B**	**Indirect effect**		**Index of mediated moderation B (95% CI)**
		**Level of pay level satisfaction**	**B (95% CI)**	
Workload ⟶ burnout ⟶ organisational turnover intention	0.153^∗^	(−1 SD)	0.304 (0.178, 0.436)	0.023 (−0.024, 0.067)
		(Mean)	0.333 (0.236, 0.438)	
		(+1 SD)	0.362 (0.254, 0.476)	

Job stress ⟶ burnout ⟶ organisational turnover intention	0.286^∗^	(−1 SD)	0.395 (0.228, 0.566)	0.032 (−0.047, 0.080)
		(Mean)	0.419 (0.288, 0.559)	
		(+1 SD)	0.442 (0.293, 0.600)	

Work–family conflict ⟶ burnout ⟶ organisational turnover intention	0.094	(−1 SD)	0.311 (0.194, 0.440)	0.016 (−0.27, 0.061)
		(Mean)	0.334 (0.238, 0.440)	
		(+1 SD)	0.356 (0.245, 0.474)	

Family–work conflict ⟶ burnout ⟶ organisational turnover intention	−0.096^∗^	(−1 SD)	0.158 (0.100, 0.225)	0.011 (−0.010, 0.034)
		(Mean)	0.172 (0.118, 0.235)	
		(+1 SD)	0.187 (0.123, 261)	

Conflict with other nurses ⟶ burnout ⟶ organisational turnover intention	0.280^∗∗^	(−1 SD)	0.208 (0.115, 0.302)	0.014 (−0.020, 0.52)
		(Mean)	0.228 (0.159, 0.302)	
		(+1 SD)	0.248 (0.170, 0.333)	

Workload ⟶ burnout ⟶ professional turnover intention	0.253^∗^	(−1 SD)	0.277 (0.134, 0.427)	0.011 (−0.041, 0.062)
		(Mean)	0.293 (0.189, 0.405)	
		(+1 SD)	0.309 (0.197, 0.419)	

Job stress ⟶ burnout ⟶ professional turnover intention	0.205	(−1 SD)	0.429 (0.234, 0.634)	0.004 (−0.070, 0.074)
		(Mean)	0.434 (0.285, 0.591)	
		(+1 SD)	0.439 (0.276, 0.602)	

Work–family conflict ⟶ burnout ⟶ professional turnover intention	0.131	(−1 SD)	0.302 (0.164, 0.441)	0.004 (−0.048, 0.053)
		(Mean)	0.308 (0.201, 0.414)	
		(+1 SD)	0.315 (0.195, 0.428)	

Family–work conflict ⟶ burnout ⟶ professional turnover intention	−0.068	(−1 SD)	0.159 (0.095, 0.232)	0.005 (−0.017, 0.029)
		(Mean)	0.165 (0.110, 0.229)	
		(+1 SD)	0.172 (0.111, 0.241)	

Conflict with other nurses ⟶ burnout ⟶ professional turnover intention	0.065	(−1 SD)	0.192 (0.82, 0.310)	0.005 (−0.035, 0.046)
		(Mean)	0.199 (0.121, 0.282)	
		(+1 SD)	0.206 (0.126, 0.288)	

Abbreviations: CI, confidence intervals; SD, standard deviation.

^∗∗^ *p*  < 0.001, ^∗^*p*  < 0.05.

## Data Availability

The data that support the findings of this study are available from the corresponding author upon reasonable request.
